# Dental home care in dogs - a questionnaire study among Swedish dog owners, veterinarians and veterinary nurses

**DOI:** 10.1186/s12917-020-02281-y

**Published:** 2020-03-18

**Authors:** Karolina Brunius Enlund, Carl Brunius, Jeanette Hanson, Ragnvi Hagman, Odd Viking Höglund, Pia Gustås, Ann Pettersson

**Affiliations:** 1 Department of Clinical Sciences, Swedish University of Agricultural Sciences, Uppsala, Sweden; 2 Anicura Albano Animal Hospital, Stockholm, Sweden; 3 Department of Biology and Biological Engineering, Food and Nutrition Science, Chalmers University of Technology, Gothenburg, Sweden

**Keywords:** Attitudes, Opinions, Practices, Canine, Survey, Tooth brushing, Veterinary dentistry, Recommendation

## Abstract

**Background:**

Periodontal disease remains one of the dog’s most common health issues, even though it is largely preventable by tooth brushing. Implementation of daily tooth brushing would not only improve animal welfare, but also reduce veterinary costs for the owner. There is a paucity of studies investigating attitudes, opinions and practices of dog owners, veterinarians, and veterinary nurses regarding preventative dental home care in dogs. The objective of this study was to investigate these parameters in Sweden, thereby providing a basis for improved prophylactic strategies.

**Methods:**

Validated questionnaire surveys were distributed to all Swedish dog owners (*n* = 209,263), veterinarians (*n* = 3657) and veterinary nurses (*n* = 1650) with e-mail addresses in the national registry. The response rates were 32% for dog owners and veterinarians, and 38% for veterinary nurses. The survey questions concerned attitudes, opinions and practices regarding dental home care, including whether dog owners received information concerning dental home care or not, and if this information resulted in implementation.

**Results:**

Attitudes, opinions and practices regarding dental home care are presented for Swedish dog owners, veterinarians, and veterinary nurses. A fundamental finding was that the absolute majority of Swedish dog owners do not perform adequate prophylactic dental home care. Considerable discrepancies were identified in the opinions of veterinary health practitioners and dog owners regarding attitudes towards dental home care and conveying of information. Several areas for improvement in the communication between dog owners and veterinary health practitioners concerning dental home care were identified.

**Conclusion:**

Our results illustrates the need for validated methods to increase dog owner compliance with dental home care recommendations. We also see a need of further education, regarding canine dental home care, among veterinarians, veterinary nurses, and dog owners. The results from this unique study constitute an important foundation for future development of prophylactic strategies, with the ultimate goal to improve dental health, and thereby animal welfare, in dogs.

## Background

Periodontal disease is the most common disease in dogs over 3 years of age, with a reported prevalence ranging between 80 and 89% [1–5]. Despite the high prevalence, the disease is considered to be severely under-diagnosed and therefore undertreated in many dogs [6]. Periodontal disease is characterized by an inflammatory chronic loss of dental supportive tissues, which may eventually result in tooth loss [3]. Studies have shown a positive correlation between severity of the disease and age. Furthermore, dogs of smaller size generally develop periodontal disease at an earlier age compared to larger dogs [1, 7, 8]. Periodontal disease should not be regarded as an isolated disease of the oral cavity but as a potential systemic disease. Associations between periodontal disease and other diseases such as renal, hepatic and cardiac diseases have been identified [6, 9–11], although not all studies are in agreement [12].

In dogs, as in humans, daily tooth brushing is considered the gold standard for prophylaxis and prevention of periodontal disease progression [13–17]. Previous studies on beagle dog colonies have provided evidence suggesting that tooth brushing three times a week might be sufficient for maintaining dental health in beagles with clinically healthy gingiva. However, in beagles with gingivitis, daily brushing was required [14, 15]. Recommended brushing frequency may thus be based on base-line oral status; however, since it cannot be predicted with certainty which individuals are more susceptible to periodontal disease, daily brushing is considered to be the gold standard [6]. While gingivitis is a reversible condition, attachment loss caused by periodontitis is considered irreversible [6]. For the dog’s well-being, prevention of disease is superior to treatment of already established disease. The objective for both dog owners and veterinary health practitioners should therefore, reasonably, be to prevent the manifestation of periodontal disease.

Tooth brushing in dogs requires owner compliance. It is well known that compliance with prescribed medical regimes is low, and often over-estimated by veterinarians [18]. In human medicine, typical compliance rates to medical treatment are only 50%, and implementation of recommended lifestyle and behavioural changes are usually even less successful [19]. However, 85% of the human population in Sweden brush their own teeth twice daily [20], and studies have shown that the quality of information and communication skills of medical personnel are crucial for the successful implementation of dental home care [21, 22]. Although the number of dog owners who brush their pets’ teeth is unknown, it is generally assumed that owner compliance with canine dental home care is inadequate. Veterinarians’ and veterinary nurses’ (Registered Veterinary Technician, RVT) strategies for conveying information regarding dental care may have a major impact on dog owner compliance. However, there is a paucity of studies regarding how dog owners receive and implement information concerning dental home care from veterinary health practitioners. In addition, studies on whether and how owners perform oral prophylactic home care in dogs are lacking.

The use of validated questionnaire surveys is a well-established method for investigating attitudes and practices [23, 24]. The aim of this study was to investigate attitudes, opinions and practices regarding dental home care in dogs, among dog owners (DO), veterinarians (V), and veterinary nurses (VN), thereby providing the basis for improved prophylactic strategies. To our knowledge, this is the first survey presented with this objective.

## Results

All recorded background characteristics of dog owners and their dogs, and for veterinarians and veterinary nurses, are summarized in supplementary information (S1 Tables).

The dogs (*n* = 59,978) were 4.9 ± 3.5 years of age (mean ± SD). All breed groups were represented. Group 8 (Retrievers, Flushing Dogs, Water Dogs) was the largest (18%), followed by dogs of mixed breed (15%) and Group 9 (Companion and Toy Dogs) (15%). One-third (33%) of dogs weighed less than 10 kg and the majority (78%) of all dogs were intact.

Dog owners (*n* = 59,978) were 49.9 ± 13.4 years of age. The majority (75%) were women, and almost half (46%) of all dog owners lived in urban counties (Stockholm, Skåne, Västra Götaland). More than two-thirds (70%) were employed or self-employed. Half (49%) had studied at a university, and almost one in four (23%) reported that they worked within a healthcare profession. Moreover, one in 12 (8%) was a dog breeder.

Veterinarians (*n* = 1114) were 42.4 ± 12.8 and veterinary nurses (*n* = 609) were 40.8 ± 9.6 years old. One-third (34%) of veterinarians and one in 12 (8%) veterinary nurses had received their degree prior to 2000. Three in four (77%) veterinarians and almost all (97%) veterinary nurses were women. Six in 10 (62%) veterinarians and half (51%) of veterinary nurses lived in an urban county (Stockholm, Skåne, Västra Götaland). Six in 10 (62%) veterinarians and nine in 10 (89%) veterinary nurses often encountered dogs in their professional role. Almost three in four (73%) veterinarians and almost all (96%) veterinary nurses worked in a pet clinic or animal hospital for dogs, cats and smaller animals, and of those, one-quarter (26%) of veterinarians and two-thirds (36%) of veterinary nurses worked at a clinic with 11 or more employed veterinarians.

**Fig. 1:**
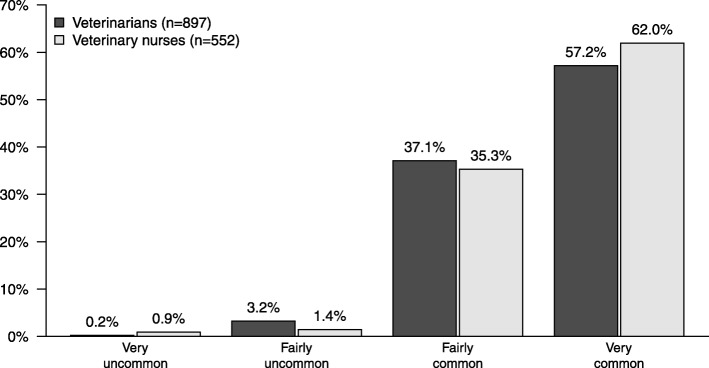
How common or uncommon do you consider dental problems to be in the dogs you meet? (V and VN) Problems include calculus, gingivitis, gum disease, tooth fractures and other dental diseases and injuries

Survey responses are summarized in supplementary information (S1 Tables). About six in 10 veterinarians and veterinary nurses stated that they very often encountered dental problems (Fig. 1), and about one in four stated they very often encountered periodontal disease (S1 Tables).

Less than one-third (29%) of dog owners, two-thirds (66%) of veterinarians and four out of five (80%) veterinary nurses consider tooth brushing very important for good dental health in dogs (S1 Tables). Most likely (respondents > 100/breed, *n* = 120) to consider tooth brushing very important for good dental health were owners of Italian Greyhound (60%), Toy Poodle (58%), Maltese (55%), Miniature Schnauzer (53%), and Yorkshire Terrier (52%). Least likely to consider tooth brushing very important for good dental health were owners of Finnish Hound (4%), Swedish Elkhound (7%), and Norwegian Elkhound (8%).

**Fig. 2:**
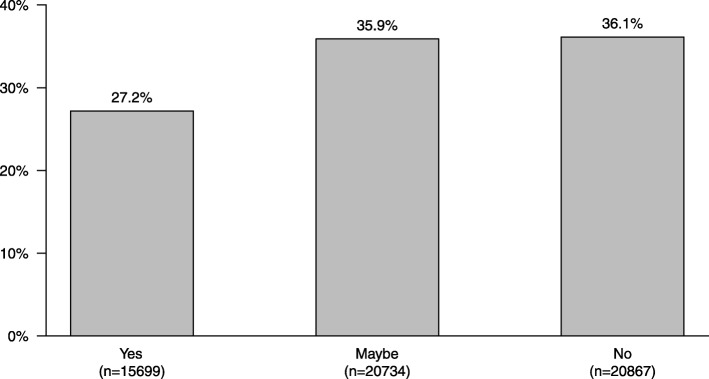
Would you consider brushing your dog’s teeth daily? (DO) (Not visible to respondents who answered that they already brush daily)

Dental cleaning with textiles was considered important for good dental health by 36% of dog owners, dog food made especially for dental health was regarded to be important by 64% of owners, and dental chews (made especially for dental health) was thought to be important by 51% of dog owners in the study (S1 Tables). Dog owners as a group stated that natural chews (e.g. rawhide) were more important for good dental health compared to tooth brushing (72% vs 61% stated importance) (χ^2^-test *p*-value < 2.2 * 10^− 16^). Almost two-thirds of dog owners stated that they could consider brushing their dog’s teeth daily (Fig. 2).

**Fig. 3:**
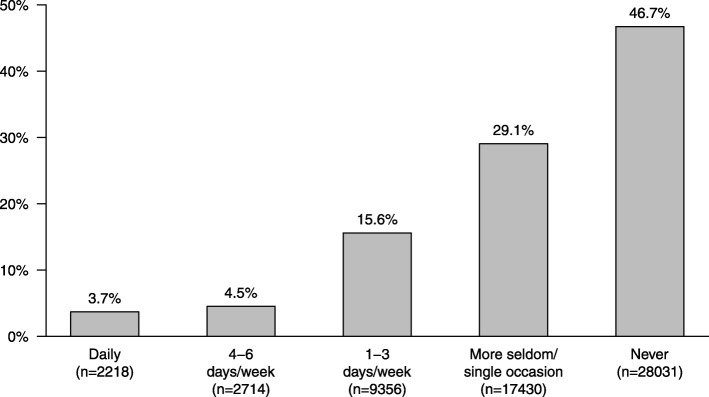
How often, in the last month, have you brushed your dog’s teeth with a toothbrush? (DO)

Less than 4 % of all dog owners brushed their dog’s teeth daily (Fig. 3). Two percent of owners used textile (e.g. finger cloth, microfiber, other textiles or gauze) 46 days/week or daily. Fourteen percent of dogs received commercial dental chews, and 20 % received natural chews (e.g. rawhide) daily (S1 Tables). The breeds (respondents > 100, *n* = 120) most likely to have their teeth brushed 46 days/week or daily were Toy Poodle (24%), Miniature Schnauzer (22%), Coton de Tuléar (21%), Miniature Poodle (21%), and Norfolk Terrier (21%). The breeds least likely to have their teeth brushed 46 days/week or daily were Finnish Hound (0.5%), German Hunting Terrier (0.6%), and Norwegian Elkhound (0.8%). One in seven (15%) dog owners stated that either they or another non-professional had on several occasions used a dental scaler to remove calculus from their dog’s teeth (S1 Tables).

Almost nine out of 10 veterinarians and veterinary nurses stated that they often or always recommend tooth brushing (Table 2). For dog owners, the most common sources of information about tooth brushing in dogs were books or journals (60%), or the internet (51%) (S1 Tables). Four in 10 (43%) dog owners stated that they had received recommendations to brush their dog’s teeth at a veterinary clinic (Table 1). Most likely (respondents > 100/breed, *n* = 120) to state that they had received recommendations at a veterinary clinic to brush were owners of Toy Poodle (74%), Yorkshire Terrier (70%), Miniature Schnauzer (68%), Coton de Tuléar (67%), and Miniature Poodle (67%). Least likely to state receiving recommendations at a veterinary clinic to brush were Norwegian Elkhound (16%), Swedish Elkhound (19%), and Hamilton Hound (21%). More than one in four veterinarians and veterinary nurses recommended a dental cleaning frequency other than daily, e.g. every other day or once a week. Information about tooth brushing was most commonly presented, according to the veterinarians and veterinary nurses, in conjunction with a visit for dental cleaning (calculus removal) or a visit due to dental problems (Table 2). One in five dog owners that had received recommendations to brush at the veterinary clinic complied with the recommendations, initiated and also continued to brush. More than one in three stated they already brushed before the recommendations (frequency of brushing unknown) (Table 1).

**Table 1:** Recommendations regarding tooth brushing (DO)

Has it ever been recommended to you by a veterinary clinic to brush/clean your dog’s teeth? Brush refers to brush with toothbrush. Cleaning refers to cleaning with textiles, e.g. finger cloth, microfiber, other textiles or gauze. (Q8)	Yes	25,337 (43.1%)
	No	31,364 (53.4%)
	Don’t know	2067 (3.5%)
When you received the recommendation at the veterinary clinic, did the information lead you to initiate brushing/cleaning your dog’s teeth? (Q13) *(only visible to the 25,337 respondents who answered that they had been recommended to do so by a veterinary clinic on question 8)*	I brushed/cleaned before I received the recommendation	9105 (35.9%)
	Yes, I brush/clean still	5124 (20.2%)
	Yes, I started (or tried) to brush/clean but stopped later	6562 (25.9%)
	No	4199 (16.6%)
	Don’t know	380 (1.5%)
When you received the recommendation at the veterinary clinic to brush/clean your dog’s teeth, what was your primary reason for the visit? Several options can be specified (Q11) *(only visible to the 25,337 respondents who answered that they had been recommended to do so by a veterinary clinic on question Q8)*	Puppy vaccination	4459 (17.6%)
	Other routine visit (e.g. vaccination)	10,803 (42.6%)
	Visit for dental cleaning (calculus removal)/dental problems	6026 (23.8%)
	Visit due to other disease	3192 (12.6%)
	Special information meeting	607 (2.4%)
	Don’t know/Other	3021(11.9%)
When you received the recommendation at the veterinary clinic to brush/clean your dog’s teeth, how did you receive the information? Several options can be specified (Q12) *(only visible to the 25,337 respondents who answered that they had been recommended to do so by a veterinary clinic on question Q8)*	Verbally	23,663 (93.4%)
	Written	1473 (5.8%)
	Practical demonstration	1761 (7.0%)
	Information about web page or similar	367 (1.4%)
	Don’t know/Other	905 (3.6%)

Dog owners’ experiences of receiving tooth brushing recommendations at a veterinary clinic

**Table 2:** Recommendations regarding tooth brushing (V and VN)

		V	VN
Do you recommend that dog owners use tooth brushing to improve the dog’s dental health? (Q11)	No, never	31 (3.5%)	4 (0.7%)
	Yes, sometimes	90 (10.1%)	31 (5.6%)
	Yes, often	198 (22.3%)	125 (22.7%)
	Yes, always	553 (62.3%)	388 (70.5%)
	Don’t know	15 (1.7%)	2 (0.4%)
When do you provide information about dental cleaning (with toothbrush or textiles) to dog owners? Several options can be specified (Q14) *(only visible to the 841 (V) and 544 (VN) respondents who answered that they recommend owners to brush/clean the dogs teeth)*	Puppy vaccination	364 (44.2%)	316 (58.6%)
	Other routine visit (e.g. vaccination)	581 (70.5%)	426 (79.0%)
	Visit for dental cleaning (calculus removal)	631 (76.6%)	448 (83.1%)
	Visit due to dental problems	655 (79.5%)	426 (79.0%)
	Visit due to other disease	421 (51.1%)	193 (35.8%)
	Special information meeting	42 (5.1%)	75 (13.9%)
	Don’t know/Prefer not to answer/Other	84 (10.2%)	40 (7.4%)
How do you provide information about dentalcleaning (with toothbrush or textiles) to dog owners? Several options can be specified (Q15) *(only visible to the 841 (V) and 544 (VN) respondents who answered that they recommend owners to brush/clean the dogs teeth)*	Verbally	789 (96.2%)	520 (96.7%)
	Written	191 (23.3%)	181 (33.6%)
	Practical demonstration	258 (31.5%)	253 (47.0%)
	Information about web pages or similar	53 (6.5%)	38 (7.1%)
	Don’t know/Prefer not to answer/Other	20 (2.5%)	16 (3.0%)
What home dental cleaning frequency doyou recommend? (Q16) *(only visible to the 841 (V) and 544 (VN) respondents who answered that they recommend owners to brush/clean the dogs teeth)*	Daily	570 (69.6%)	402 (74.9%)
	Every other day	40 (4.9%)	25 (4.7%)
	Once a week	50 (6.1%)	12 (2.2%)
	As often as they have time for	75 (9.2%)	64 (11.9%)
	I don’t specify	29 (3.5%)	10 (1.9%)
	Don’t know/Prefer not to answer/Other	55 (6.7%)	24 (4.5%)
Do you follow up whether the dog owner is satisfactorily performing dental home care on the dog? Follow up means checking if the dog owner is carrying out dental home care on the dog, e.g., via telephone call, email, visit or re-visit. (Q17)	No, never	332 (38.2%)	196 (36.1%)
	Yes, sometimes	391 (45.0%)	262 (48.3%)
	Yes, often	76 (8.8%)	54 (9.9%)
	Yes, always	10 (1.2%)	8 (1.5%)
	Don’t know/Prefer not to answer	60 (6.9%)	23 (4.2%)

Veterinarians’ (V) and veterinary nurses’ (VN) recommendation routines regarding canine tooth brushing

Two-thirds (67 and 64% respectively) of dog owners stated that important reasons for tooth brushing was for maintaining the dog’s teeth, and for good general health in the dog. Half of the veterinarians (51%) and veterinary nurses (56%) stated that they consider lack of time to be a common reason for veterinary health practitioners to not talk about tooth brushing. Veterinarians (40%) and veterinary nurses (52%) considered owners lack of knowledge to be a common reason why dog owners do not brush their dog’s teeth (S1 Tables). About one in 10 veterinarians and veterinary nurses state that they often or always performed follow-ups on dental home care (Table 2).

**Fig. 4:**
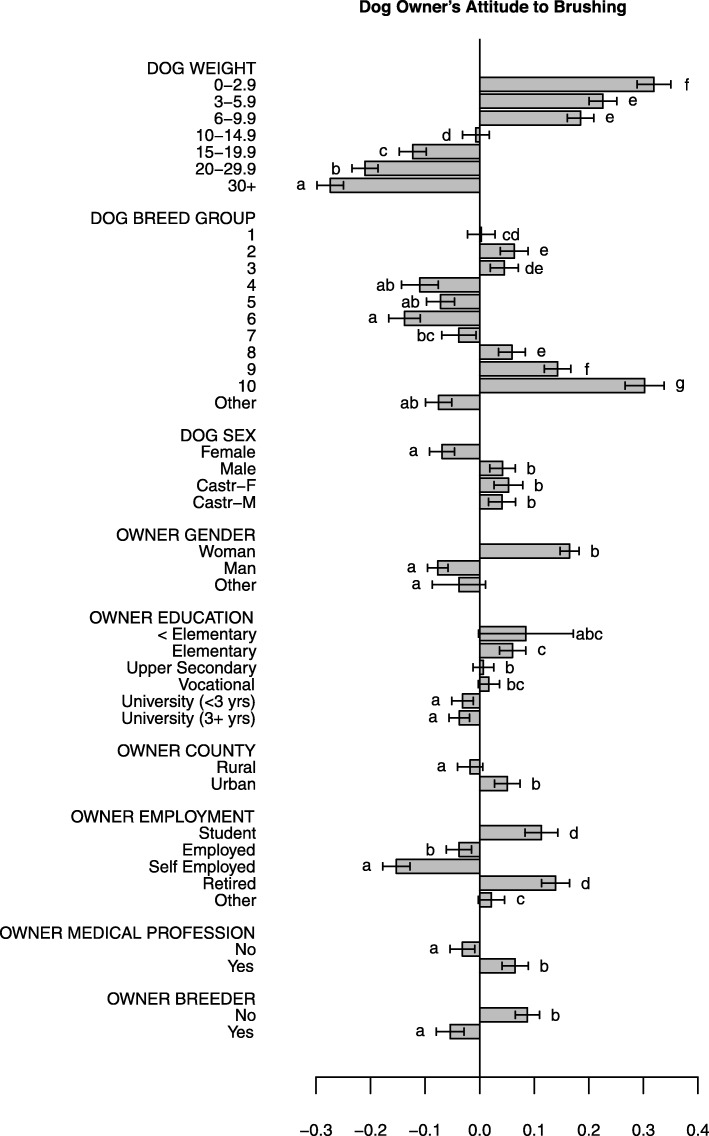
Construct “Dog owners’ attitudes towards brushing dogs’ teeth” *(BrushAttitude)*. Associations between background characteristics of dog/dog owner, and dog owners’ attitude towards tooth brushing in the dog, where a higher construct score reflects a more positive attitude towards canine tooth brushing. Scores should only be compared within figure. (Note that negative scores do not automatically reflect a negative attitude towards brushing)

Associations between background characteristics of dog/dog owner, and dog owners’ attitude towards tooth brushing in the dog are shown in Fig. 4.

**Fig. 5:**
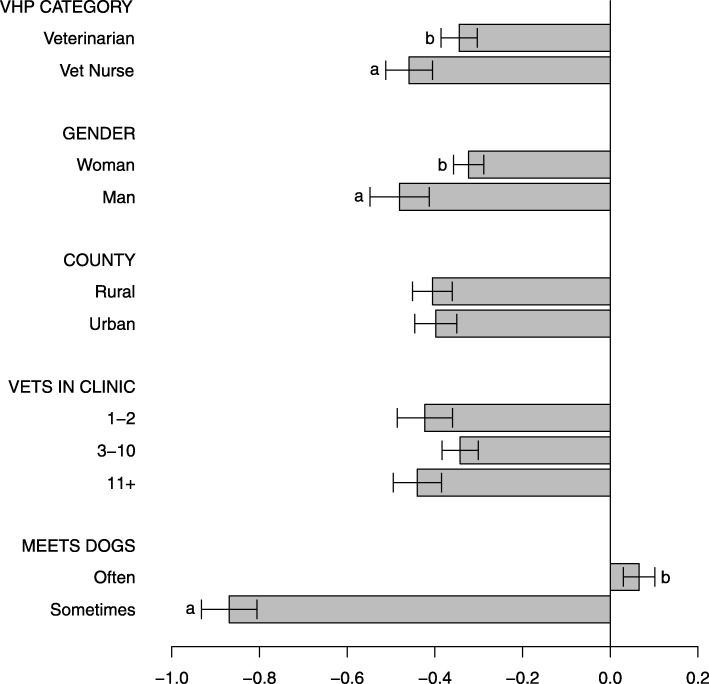
Construct “Veterinary health practitioners’ attitudes and opinions on dental problems and dental cleaning” *(Cleaning)*. Associations between background characteristics of veterinarians and veterinary nurses, and their attitudes and opinions on dental problems and dental cleaning/tooth brushing. A higher construct score reflects the experience of dental problems and periodontal disease as more common, as well as recommending tooth brushing more often, i.e. a more positive attitude towards cleaning/tooth brushing. Scores should only be compared within figure. (Note that negative scores do not automatically reflect a negative attitude towards dental cleaning/tooth brushing)

Associations between background characteristics of veterinarians and veterinary nurses, and their attitudes and opinions on dental problems and dental cleaning/tooth brushing are shown in Fig. 5.

Smaller dogs were more likely to have their teeth brushed more often, and owners of smaller dogs were more likely to report that they received recommendations to brush at a veterinary clinic. Dogs that received dental chews also had their teeth brushed more often. Odds ratios for the influence of dog owners’ background characteristics on brushing frequency and their perception of receiving recommendations at the veterinary clinic to brush their dog’s teeth, and the influence of administration of dental chews on brushing the dog’s teeth, are shown in S1 Figures.

Older dog owners and owners of younger dogs had a more positive attitude towards brushing their dogs’ teeth than their counterparts (S1 Figures).

Veterinarians and veterinary nurses who often meet dogs were more likely to state tooth brushing to be important for good dental health, and they had a more negative attitude towards dental chews and dental feed than their counterparts. Odds ratios for the influence of veterinarians’ and veterinary nurses’ background characteristics on stating tooth brushing to be important for good dental health in dogs are shown in S1 Figures. Associations between background characteristics of the veterinary health practitioner and their attitude towards dental chews and dental feed are shown in S1 Figures.

Veterinary health practitioners with a more recent year of degree had a more positive attitude towards dental cleaning/tooth brushing, and a more negative attitude towards dental chews/dental feed, than their counterparts (S1 Figures).

## Discussion

In this study, we present attitudes, opinions and practices of dog owners, veterinarians, and veterinary nurses regarding preventative dental home care in dogs, thereby providing a basis for future prophylactic strategies.

### Opinions on dental health

The majority of veterinarians and veterinary nurses in the present study perceived dental problems to be very common (Fig. 1). This is in agreement with a previous commercial survey among veterinarians and veterinary nurses in UK 2012, where 60% of respondents estimated that at least three in five dogs over 3 years of age suffered from periodontal disease [25]. In another study among Dutch veterinarians, 97% of the respondents stated that they regularly observed periodontal problems in their canine patients [26]. The high awareness of dental problems in dogs among Swedish veterinarians and veterinary nurses is also in line with the high prevalence of dental disease reported in other studies [1–4, 6].

### Attitudes and opinions on dental home care in the dog

The majority of all respondents reported that dental home care in some form was indeed important. Therefore, all comparisons of attitudes of subgroups of respondents, and relative differences should be seen as degrees of a generally positive attitude towards canine dental home care. In the sample (and likely in the target population) there are systematic associations, e.g. more women own small dogs. However, this has been adjusted for in the statistical models (Figs. 4 and 5, S1 Figures).

Only 29% of dog owners rated tooth brushing to be very important for good dental health. There was a strong association between dog owners’ attitudes towards brushing their dog’s teeth and the dog’s weight: the smaller the dog, the more positive the attitude towards brushing (Fig. 4). This likely reflects the higher prevalence of periodontal disease seen in smaller dogs [7]. Dog owners who worked in a health-related profession had a more positive attitude to brushing (Fig. 4), probably a result of greater knowledge concerning dental disease. Interestingly, women had a more positive attitude towards tooth brushing than men, and students and pensioners were overall more positive towards tooth brushing than the other subgroups (Fig. 4). Although education, owner age, dog age, county, and dog sex were associated with attitude to brushing, the effect sizes were smaller and should therefore be interpreted cautiously (Fig. 4, S1 Figures). Discouragingly, dog owners as a group stated that natural chews (e.g. rawhide) were more important for good dental health than tooth brushing (*p* < 2.2 * 10^− 16^) (S1 Tables). There was thus a clear discrepancy in the attitude to tooth brushing between dog owners, illustrating a knowledge gap between the groups. Encouragingly, however, the majority of dog owners who currently did not brush daily, answered that they might consider doing so (Fig. 2), indicating large potential motivation to perform dental home care, given proper support and information.

In contrast to dog owners’ attitudes, 66% of the veterinarians and 80% of the veterinary nurses rated brushing to be very important (S1 Tables). The *Cleaning*-construct indicated that female practitioners, those who graduated more recently, and those who met dogs more often perceived dental problems and periodontal disease as more common and recommended tooth brushing more often, compared to their counterparts (Fig. 5, S1 Figures). On the other hand, male practitioners, those who graduated earlier, and veterinarians or veterinary nurses that only occasionally met dogs were more positive in their attitudes towards dental chews and dental feed (S1 Figures). According to WSAVA’s dental guidelines, this kind of passive dental home care generally has limited effect on dental health, and should be seen as a supplement to active home care, i.e. tooth brushing [6]. These findings highlight differences in knowledge and attitudes among practitioners and indicate where educational efforts could have the highest impact for improved canine dental health.

### Dental home care practices

Approximately half of the dog owners in this survey never brushed their dog’s teeth and almost one-third brushed less frequently than once a week, or only on single occasions (Fig. 3). Despite daily brushing being the gold standard for prevention of dental problems, less than 4% of dog owners brushed daily (Fig. 3) [13, 27]. Considering social desirability bias and that respondents participating in the survey may have been initially more interested in the subject [28], even this low number may in fact constitute an over-estimation of brushing frequency. Although the frequency of tooth brushing in a dog population has previously never been reported in the scientific literature, a Canadian market research company has investigated the frequency of tooth brushing in Canadian dogs and reported similar results [29].

In line with owners of smaller dogs having a more positive attitude towards tooth brushing, we observed that owners of smaller dogs also were prone to brush more frequently (S1 Figures). This likely reflects that the higher prevalence of periodontal disease reported for smaller breeds [7] has resulted in an increased awareness of dental health among their owners. Differences between breeds in frequency of brushing may also be caused by differences in owner characteristics or breed-related dog behaviour and subsequent manageability. In addition, the likelihood of high brushing frequency was associated with dog breed group. Surprisingly, owners of sight hounds (breed group 10) were the most likely to brush their dog’s teeth (S1 Figures). The reasons for this remains to be elucidated.

Although brushing every other day may be sufficient to maintain dental health in dogs with clinically healthy gingiva [14], daily brushing may be required for ensuring dental health, especially for at-risk-individuals [14, 15]. Our results indicate that this fact may not be common knowledge among dog owners. Further, the majority of dog owners used natural chews (e.g. rawhide), and almost half used dental chews at least once a week (S1 Tables), which is comparable with an Italian study where 55% of dog owners used dental care sticks [30]. Natural chews may be a way of activating the dog, but their effect on dental health has, to the authors’ knowledge, not been investigated [27]. Dental chews and feeds, on the other hand, are used primarily to promote dental health. However, the quality and efficacy of dental chews vary widely: Some have undergone clinical trials with significant results on plaque and calculus under experimental conditions [31], whereas others have as yet no proven effect. Still, daily tooth brushing remains the most effective way to minimize dental plaque. A recent study showed that daily tooth brushing was more than three times as effective at controlling plaque accumulation compared to the use of a daily dental chew or dental diet [32]. The use of dental chews was directly associated with the frequency of tooth brushing (data not shown), indicating that dog owners with a higher interest in dental home care are users of both passive and active dental home care. However, the observed knowledge gaps may contribute to a false sense of security for dog owners who brush regularly but less often, or who rely on passive dental care.

One-fifth of dog owners stated that a non-professional had used an instrument to remove dental calculus from their dog’s teeth above the gingival margin. In addition to potentially damaging the tooth enamel, the method is purely cosmetic and does not protect against periodontal disease, since it does not clean below the gum line [6]. Our results indicate that many dog owners may not be aware of this (S1 Tables).

### Recommendations on dental home care in the dog

The vast majority of veterinarians and veterinary nurses in this study stated that they often or always recommended tooth brushing (Table 2). This is in stark contrast to what the dog owners report. In fact, more than half of the dog owners stated that they had never received recommendations at the veterinary clinic to brush their dog’s teeth (Table 1), although most Swedish dog owners visit veterinary clinics regularly, e.g. for routine vaccination. There may be several reasons for this discrepancy; for instance, dog owners may not remember receiving recommendations. In support of this hypothesis, a previous study showed that although almost all dog owners recalled receiving verbal information, 33% could not recall observing a practical demonstration of dog tooth brushing, and 8% did not remember receiving tooth brushing equipment, when interviewed at follow-ups after 13 months [33]. Another likely reason for the discrepancy between dog owners’ and professionals’ perceived recommendations of brushing may be that the veterinarians and veterinary nurses who answered the surveys may have been extra interested in veterinary dentistry and therefore more prone to participate.

Owners of smaller dogs and Companion and Toy Dogs (breed group 9) were more likely to report having received recommendations on tooth brushing at the veterinary clinic (S1 Figures). This likely reflects veterinary health practitioners’ aforementioned higher awareness of dental problems for smaller breeds [1, 7, 8]. Interestingly, women were more likely than men to answer that they had been recommended to brush (S1 Figures), which may indicate the need for more targeted efforts towards male dog owners.

Of those who recommended tooth brushing, seven out of 10 veterinarians and three out of four veterinary nurses recommended tooth brushing once a day. The remaining veterinarians and veterinary nurses recommended a different brushing frequency, e.g. every other day, once a week or “as often as possible”, or did not specify frequency at all (Table 2). Although the results show that the awareness regarding the need for brushing daily is fairly high, there is still room for improvement.

The most common occasions when veterinarians and veterinary nurses discussed tooth brushing with dog owners were in conjunction with visits for dental problems, dental cleaning and booster vaccinations (Table 2). This is in line with the results of a commercial survey among the UK’s vets and nurses from 2012 [25]. Additionally, several veterinarians and veterinary nurses commented in the free-text that they recommended tooth brushing “when a problem is seen”, “when needed” or “when dental calculus is seen”, which must be deemed too late in the course of the disease [6]. Some veterinarians and veterinary nurses commented in free text that they more often recommended tooth brushing to owners of small breeds, because they considered home care to be of more importance for these breeds, likely reflecting the aforementioned higher prevalence of periodontal disease in smaller breeds. Almost half of the veterinarians and veterinary nurses stated that they recommended tooth brushing at the puppy vaccination, whereas less than one-fifth of dog owners stated that they received information on this occasion (Tables 1 and 2). Although the populations are not matched, these results clearly illustrate a communication gap. The visit to the veterinary clinic for puppy vaccination presents an ideal opportunity to influence new dog owners to initiate proper prophylactic tooth brushing routines.

**Table 3:** Details of questionnaire recipients and responses

	Target population	Recipients	Total number of respondents	Total number of completed responses^a^
Dog owners^b^	607,610	209,263	66,434 (32%)	59,978
Veterinarians	4081	3657	1161 (32%)	1114
Veterinary nurses	1814	1650	642 (38%)	609

Number of individuals in target populations (dog owners, veterinarians, and veterinary nurses), questionnaire recipients and survey respondents before and after removing respondents with missing data

^a^ After removing individuals with > 20% missing data among selected background questions

^b^ Out of the dog owners in the target population, 23% owned more than one dog (personal communication, Magnus Kindström, Swedish Board of Agriculture, 28 August 2017)

**Table 4:** Questions related to the constructs “*BrushAttitude*” and “*Cleaning*”

“Dog owners’ attitudes towards brushing dogs’ teeth” (*BrushAttitude*) (Cronbach’s alpha = 0.86)	
How often in the last month have you brushed your dog’s teeth with a toothbrush? [14]	
How often in the last month have you used dog tooth paste on your dog? [21]	
Do you consider tooth brushing to be important for good dental health in the dog? [7]	
Do you consider dog toothpaste to be important for good dental health in the dog? [7]	
Would you consider brushing your dog’s teeth daily? [16]	
“Veterinary health practitioners’ attitudes and opinions on dental problems and dental cleaning” *(Cleaning)* (Cronbach’s alpha 0.73)	
Do you recommend that dog owners use tooth brushing to improve the dog’s dental health? [11]	
What dental cleaning frequency do you recommend? [16]	
Do you consider tooth brushing to be important for good dental health in dogs? [8]	
Do you recommend that dog owners use dental cleaning with textiles (e.g. finger cloth, microfiber, cloth or gauze) to improve the dog’s dental health? [11]	
Do you recommend that dog owners use mouthwash or mouth gel with chlorhexidine to improve the dog’s dental health? [11]	
How common or uncommon do you consider gum disease (periodontal disease) to be in the dogs you meet? [10]	
How common or uncommon do you consider dental problems to be in the dogs you meet? [9]	

Number in parenthesis after the question corresponds to the number of the question in the full survey. Adapted from [28]

Interestingly, of those dog owners who reported that they had been recommended at the veterinary clinic to brush, as many as one in four had initiated but discontinued tooth brushing (Table 1). Similar results have been reported previously [33]. This further highlights the need for improvement in the supportive systems provided to dog owners by the veterinary clinic, regarding dental home care.

Although veterinary nurses in Sweden often spend more time with dog owners (telephone, reception, examination room) than veterinarians, dog owners report that veterinary nurses seldom (only in 15% of cases) provided information concerning tooth brushing (S1 Tables). Much as in human dentistry, where dental hygienists provide much of the prophylactic information, veterinary nurses could play an important role in building effective routines for conveying information concerning dental home care. This model may also be a way forward within veterinary dentistry.

### Reasons to perform dental home care and compliance with recommendations

In this study, we have identified different underlying factors that may increase dog owners’ motivation to perform dental home care. The most important factors for dog owners to brush their dog’s teeth were: “That the dog should keep its teeth” and “That it is good for the dog’s general health” (S1 Tables). These motivational factors should therefore be incorporated in prophylactic strategies to maximize the chances of implementation of dental home care.

While several veterinarians and veterinary nurses stated that information concerning tooth brushing may not be delivered to dog owners because the dog owner was assessed to be unable, or the dog to be totally uncooperative, a majority of dog owners in fact stated that they would consider brushing daily (Fig. 2). This suggests that veterinarians and veterinary nurses may underestimate dog owners’ willingness and capability to perform dental home care.

Only a few studies have investigated the compliance with dental home care recommendations for dogs and these were based on small study groups [33, 34]: one study reported that only half of the dog owners brushed several times a week or daily 1 year after receiving recommendations [33]. Studies in humans have shown that regular follow-up is an important determinant for compliance with medical advice, which may be accomplished by, for example counselling, support-groups, and reminders [19]. However, half of the veterinarians and veterinary nurses in this study performed follow-up on dental home care only occasionally, and over one-third never performed such follow-ups (Table 2). These results indicate that there is room for improvement with regard to follow-up routines of dental home care.

### Strengths and limitations

As previously discussed, questionnaire surveys inevitably contain bias, such as recruitment bias, social desirability bias, and acquiescence bias [28]. There is e.g. a potential risk of respondents being more interested in the subject than the average population. Using pre-formulated sentences as response options entails a risk of misinterpretation of opinions. However, a thorough validation was performed to minimize these risks, including a discussion of the surveys’ response rate [28]. The use of vague response options, such as “sometimes”/“often” or “not common, very common” for several of the survey questions poses limitations on the possibilities for quantification of actual frequencies. However, this was an informed decision based on the aims of the study, i.e. to measure attitudes and opinions. Further, the presented study was performed in a Swedish social and cultural context which should be taken into account in any comparisons [28].

The study also has several strengths: the study samples were very large and collected responses are therefore likely to accurately reflect opinions and attitudes of the study populations. In addition, the representativity of the respondents to the target population was thoroughly investigated and found to be overall satisfactory, and state-of-the-art methods in survey construction and validation were applied to ensure high data quality [28].

## Conclusion

The present study provides unique insights into attitudes and motivational factors of dog owners, veterinarians, and veterinary nurses regarding dental home care. A fundamental finding was that the absolute majority of Swedish dog owners do not perform adequate prophylactic dental home care. Discrepancies between dog owners’ and veterinary health practitioners’ attitudes towards dental home care were identified: dog owners were more positive towards passive dental home care, whereas veterinarians and veterinary nurses were more positive towards tooth brushing, the gold standard in preventative dental home care. Further, a discrepancy was exposed between the dog owners and veterinary health practitioners regarding the perception of whether and how recommendations of tooth brushing were conveyed. Lack of knowledge regarding dental care among both dog owners and veterinary health practitioners has been revealed, as well as veterinarians’ and veterinary nurses’ preconceptions concerning dog owners’ attitudes towards dental home care.

The results from this study constitute an important foundation for future development of prophylactic strategies, with the ultimate goal to improve dental health, and thereby animal welfare, in dogs.

## Methods

Two questionnaire surveys, one to dog owners and one to veterinarians and veterinary nurses, were constructed and validated according to survey methodology guidelines [28]. The study was approved by the Regional Ethical Review Board in Uppsala (Dnr 2017/035).

### Study design

Target groups consisted of all currently registered dog owners in Sweden (DO), all registered veterinarians (V), and all registered licensed veterinary nurses (VN) in Sweden.

Sample frames were dog owners, veterinarians, and licensed veterinary nurses with e-mail addresses registered with the Swedish Board of Agriculture (24 February 2017 for V and VN; 13 March 2017 for DO). Veterinarians were also contacted by text message to their mobile telephone numbers from the same register. Furthermore, for dog owners, e-mail addresses registered in the Swedish Kennel Club (9 February 2017) were used.

The questionnaire surveys were adapted for use on personal computers, tablets and smart phones, using the web platform Netigate (Netigate AB, Stockholm, Sweden). The questionnaires were distributed and reminders were sent to non-responders after eight and 17 days. Data collection started on 31 March and was completed on 30 April 2017. Anonymous responses were collected, and the questionnaire could only be answered once per link. If the household owned more than one dog, the respondent was asked to choose one and answer for the same dog throughout the survey. Details on survey administration are reported in Table 3 [28]. The length of the questionnaire for individual respondents depended on their answers and ranged from 17 to 68 questions. The questions were mainly closed, i.e. with fixed response options, and both nominal and ordinal data were collected [28].

From all data available from the questionnaires, we here present and discuss questions and constructs reflecting dental home care.

### Statistical analysis

The breeds (*n* = 316) were grouped into 10 groups as used by the Federation Cynologique Internationale (FCI) [35] as well as the Swedish Kennel Club. Statistical analyses on breeds (regarding owners perceived importance of tooth brushing, frequency of tooth brushing, and receiving recommendations at a veterinary clinic to brush) were restricted to those breeds represented by more than 100 respondents (*n* = 120) .

Pretreatment of data, including identification and validation of constructs, is described in detail elsewhere [28]. In brief, exploratory factor analysis (EFA) was performed on random half-splits of numeric and ordinal non-sociodemographic data to identify factors, which were confirmed in the other half-split using confirmatory factor analysis (CFA). Final construct scores were extracted from CFA on all data using variables selected from the EFA/CFA validation procedure. The constructs reflected core concepts regarding canine dental home care. The three constructs used in this study, i.e. “Dog owners’ attitudes towards brushing dogs’ teeth” *(BrushAttitude)*, “Veterinary health practitioners’ attitudes and opinions on dental problems and dental cleaning” (*Cleaning)*, and “Veterinary health practitioners’ attitudes towards dental chews and dental feed” (*ChewFeed*) are illustrated in Table 4 and S1 Tables.

All statistical analysis was performed in the R open source statistical software v 3.5.1 [36]. Overall significance of fixed factors in linear mixed modelling was assessed by type III tests and using Tukey adjustment for pairwise comparisons. Results are reported as least squares means with 95% CI. Results from logistic regressions are reported as odds ratios with 95% CI.

From the dog owner survey data, the *BrushAttitude* construct was analysed by linear mixed modelling using the R ‘glm’ function. Dog weight group, sex and breed group, and owner gender, level of education, county (urban vs rural), employment, medical profession or breeder status were included as fixed factors. In addition, dog and owner year-of-birth were added as covariates. The question “How often in the last month have you brushed your dog’s teeth with a toothbrush? (Q14)” was analysed by ordinal logistic regression using the R ‘polr’ function from the ‘MASS’ package and with the same fixed factors and covariates. The question “Has it ever been recommended to you by a veterinary clinic to brush/clean your dog’s teeth? (Q8)” was analysed by logistic regression using the R ‘glm’ function (family = ‘binomial’) and with the same fixed factors and covariates. Analyses of *BrushAttitude,* Q14 and Q8 were performed for all complete responders (*n* = 59,978) (Table 3). Difference in proportion of responders stating tooth brushing vs chews being important for dental health was analysed by χ^2^-test, considering *p* < 0.05 as statistically significant.

From the veterinary health practitioner survey data, the *ChewFeed* and *Cleaning* constructs were analysed for all complete responders who treated dogs in their practice (*n* = 1436) by linear mixed modelling. Fixed factors included profession (veterinarian vs veterinary nurse), gender, county (urban vs rural), size of clinic, and whether they treated dogs in their practice (sometimes vs often). In addition, year-of-degree was added as a covariate. The question “How important do you consider tooth brushing to be for good dental health in dogs? (Q8)” was analysed for all complete responders (*n* = 1725) by ordinal logistic regression using whether they treated dogs in their practice (never, sometimes or often) as fixed factor.

## Supplementary information

**Additional file 1: Table S1.** File containing supplementary tables with complete results. A. Results from the dog owner survey. B. Results from the veterinarian and veterinary nurse survey. C. Questions related to the construct “Veterinary health practitioners’ attitudes towards dental chews and dental feed” *(ChewFeed).* D. Background characteristics of dogs. E. Background characteristics of dog owners. F. Background characteristics of veterinarians and veterinary nurses.

**Additional file 2: Figure S1.** File containing supplementary figures. A. Odds ratio (95% CI) for dog owners’ background characteristics influence on brushing frequency. B. Odds ratio (95% CI) for dog owners’ background characteristics influence on perceived reception of recommendations at the veterinary clinic to brush their dog’s teeth*.* C. Odds ratio (95% CI) for veterinarians’ and veterinary nurses’ background characteristics influence on stating tooth brushing to be important to good dental health in dogs. D. Associations between background characteristics of veterinarians/veterinary nurses, and their attitude towards dental chews and dental feed. E. Odds ratio of the influence of administration of dental chews on brushing the dogs teeth. F. Construct’s “Dog owners’ attitudes towards brushing dogs’ teeth” *(BrushAttitude)* association with the year of birth of the dog and dog owner. G. Construct’s “Veterinary health practitioners’ attitudes towards dental chews and dental feed” *(ChewFeed)* and Construct’s “Veterinary health practitioners’ attitudes and opinions on dental problems and dental cleaning” *(Cleaning)* association with the year of degree of the veterinary health practitioner.

## Data Availability

The data are not publicly available since they contain information that could compromise research participant privacy, but are available from the corresponding author upon reasonable request.

## Abbreviations

*BrushAttitude*: Construct “Dog owners’ attitudes towards brushing dogs’ teeth”
CFA: Confirmatory factor analysis
*ChewFeed*: Construct “Veterinary health practitioners’ attitudes towards dental chews and dental feed”
*Cleaning*: Construct “Veterinary health practitioners’ attitudes and opinions on dental problems and dental cleaning”
DO: Dog owners
EFA: Exploratory factor analysis
V: Veterinarians
VN: Veterinary nurses
