# Supplementary material for: Dental home care in dogs - a questionnaire study among Swedish dog owners, veterinarians and veterinary nurses
Source: BMC Vet Res. 2020 Mar 18;16:90. doi: 10.1186/s12917-020-02281-y (PMC7081671; doi:10.1186/s12917-020-02281-y)
Supplement: Supplementary file 1 — Additional file 1: Table S1. File containing supplementary tables with complete results. A. Results from the dog owner survey. B. Results from the veterinarian and veterinary nurse survey. C. Questions related to the construct “Veterinary health practitioners’ attitudes towards dental chews and dental feed” (ChewFeed). D. Background characteristics of dogs. E. Background characteristics of dog owners. F. Background characteristics of veterinarians and veterinary nurses. [file 12917_2020_2281_MOESM1_ESM.docx]

**Dental home care in dogs - a questionnaire study among Swedish dog owners, veterinarians and veterinary nurses**

**Supplementary Information Tables**

**Table A. Results from the dog owner survey**. Number in parenthesis after the question corresponds to the number of the question in the full survey.

|  | | | | **Not at all important** | | **Of minor importance** | **Fairly important** | **Very important** | | **Don't know** |
| --- | --- | --- | --- | --- | --- | --- | --- | --- | --- | --- |
| **What do you consider to be important for good dental health in dogs? (7)** | **Tooth brushing** | | | 4643 (8.1%) | | 13654 (23.9%) | 18413 (32.2%) | 16572 (29.0%) | | 3948 (6.9%) |
|  | **Dental cleaning with textiles** | | | 7971 (13.9%) | | 17175 (30.0%) | 14390 (25.2%) | 6130 (10.7%) | | 11537 (20.2%) |
|  | **Dog food special for dental health** | | | 3314 (5.8%) | | 11868 (20.7%) | 21306 (37.2%) | 15157 (26.5%) | | 5589 (9.8%) |
|  | **Dental chews special for dental health** | | | 8271 (14.5%) | | 16301 (28.5%) | 19129 (33.5%) | 10068 (17.6%) | | 3365 (5.9%) |
|  | **Natural chewing bones, e.g. rawhide** | | | 2959 (5.2%) | | 10012 (17.5%) | 24958 (43.7%) | 16385 (28.7%) | | 2802 (4.9%) |
|  | **Dog toothpaste** | | | 11096 (19.4%) | | 19754 (34.6%) | 13563 (23.7%) | 6003 (10.5%) | | 6735 (11.8%) |
|  | | | **Daily** | | **4-6 days/week** | **1-3 days/week** | **More seldom/single occasion** | | **Never** | **Don't know** |
| **How often in the last month have you brushed your dog's teeth with a toothbrush? (14)** | | | 2218 (3.7%) | | 2714 (4.5%) | 9356 (15.6%) | 17430 (29.1%) | | 28031 (46.7%) | 229 (0.4%) |
| **How often in the last month have you cleaned your dog's teeth with textiles (e.g. finger cloth, microfiber, other textiles or gauze)? (15)** | | | 642 (1.1%) | | 734 (1.2%) | 3339 (5.6%) | 11746 (19.6%) | | 43199 (72.0%) | 318 (0.5%) |
| **How often in the last month has your dog chewed/used any of the following: (21)** | | **Dental chews special for dental health** | 8127 (13.9%) | | 4178 (7.2%) | 12811 (21.9%) | 21728 (37.2%) | | 11386 (19.5%) | 154 (0.3%) |
|  |  | **Natural chewing bones, e.g. rawhide** | 11940 (20.4%) | | 6267 (10.7%) | 16644 (28.5%) | 16366 (28.0%) | | 7095 (12.1%) | 123 (0.2%) |

|  | | **Yes** | **Maybe** | | **No** | | **Prefer not to answer** | |
| --- | --- | --- | --- | --- | --- | --- | --- | --- |
| **Would you consider brushing your dog’s teeth daily? (16)** *(Not visible to the 2218 respondents who answered “daily” on question 14)* | | 15699 (27.2%) | 20734 (35.9%) | | 20867 (36.1%) | | 462 (0.8%) | |
|  | | **No** | **Yes, once** | | **Yes, several times** | | **Don’t know** | |
| **Has a dental scaler been used by yourself or someone else (e.g. groomer or breeder) to remove the dog’s dental calculus?** Note: This does not include calculus removal at a veterinary clinic. **(22)** | | 46093 (76.8%) | 3436 (5.7%) | | 9111 (15.2%) | | 1338 (2.2%) | |
|  | | | | **Yes** | | **No** | | **Don’t know** |
| **Has it ever been recommended to you, by any of the following, to brush/clean your dog's teeth?** Brush refers to brush with toothbrush. Cleaning refers to cleaning with textiles, e.g. finger cloth, microfiber, other textiles or gauze. **(8)** | **By a veterinary clinic** | | | 25337 (43.1%) | | 31364 (53.4%) | | 2067 (3.5%) |
|  | **By breeder** | | | 13230 (22.5%) | | 42589 (72.5%) | | 2938 (5.0%) |
|  | **By breed club/dog club (e.g. member journal or lecture)** | | | 14723 (25.1%) | | 38198 (65.1%) | | 5770 (9.8%) |
|  | **By friends or family** | | | 17237 (29.4%) | | 39576 (67.4%) | | 1873 (3.2%) |
|  | **By books or journals** | | | 35202 (59.9%) | | 19120 (32.6%) | | 4398 (7.5%) |
|  | **By the internet (e.g. information pages or social media)** | | | 29712 (50.6%) | | 24440 (41.6%) | | 4573 (7.8%) |
|  | **By my own healthcare education** | | | 12035 (20.5%) | | 42088 (71.7%) | | 4606 (7.8%) |
| **Have you been advised at a veterinary clinic to use any of the following to improve your dog's dental health? (27)** | **Dog food special for dental health** | | | 4022 (6.9%) | | 51918 (89.4%) | | 2125 (3.7%) |
|  | **Dental chews special for dental health** | | | 6644 (11.4%) | | 48886 (84.2%) | | 2559 (4.4%) |
|  | **Natural chewing bones, e.g. rawhide** | | | 6113 (10.5%) | | 49096 (84.6%) | | 2820 (4.9%) |
|  | **Dog toothpaste** | | | 12499 (21.5%) | | 43108 (74.3%) | | 2424 (4.2%) |

| **When you received the recommendation at the veterinary clinic, did the information lead you to initiate brushing/cleaning your dog's teeth? (13)** *(only visible to the 25337 respondents who answered that they had been recommended to do so by a veterinary clinic on question 8)* | I brushed/cleaned before I received the recommendation | 9105 (35.9%) |
| --- | --- | --- |
|  | Yes, I brush/clean still | 5124 (20.2%) |
|  | Yes, I started (or tried) to brush/clean but stopped later | 6562 (25.9%) |
|  | No | 4199 (16.6%) |
|  | Don't know | 380 (1.5%) |
| **When you received the recommendation at the veterinary clinic to brush/clean your dog's teeth, what was your primary reason for the visit? Several options can be specified (11)** *(only visible to the 25337 respondents who answered that they had been recommended to do so by a veterinary clinic on question 8)* | Puppy vaccination | 4459 (17.6%) |
|  | Other routine visit (e.g. vaccination) | 10803 (42.6%) |
|  | Visit for dental cleaning (calculus removal)/dental problems | 6026 (23.8%) |
|  | Visit due to other disease | 3192 (12.6%) |
|  | Special information meeting | 607 (2.4%) |
|  | Don't know/Other | 3021(11.9%) |
| **When you received the recommendation at the veterinary clinic to brush/clean your dog's teeth, how did you receive the information? Several options can be specified (12)** *(only visible to the 25337 respondents who answered that they had been recommended to do so by a veterinary clinic on question 8)* | Verbally | 23663 (93.4%) |
|  | Written | 1473 (5.8%) |
|  | Practical demonstration | 1761 (7.0%) |
|  | Information about web page or similar | 367 (1.4%) |
|  | Don't know/Other | 905 (3.6%) |
| **When you received the recommendation at the veterinary clinic to brush/clean your dog's teeth, who gave you the information? (10)** *(only visible to the 25337 respondents who answered that they had been recommended to do so by a veterinary clinic on question 8)* | The veterinarian | 12603 (49.5%) |
|  | The veterinary nurse/animal caretaker | 3865 (15.2%) |
|  | Both the veterinarian and the veterinary nurse/animal caretaker | 6747 (26.5%) |
|  | Don't know/Other | 2279 (8.9%) |
| **Do you as a breeder recommend that puppy buyers brush their dog's teeth? (32)** *(Only visible to the 5030 respondents who answered that they were breeders)* | No, never | 1648 (32.7%) |
|  | Yes, sometimes | 1129 (22.4%) |
|  | Yes, often | 530 (10.5%) |
|  | Yes, always | 1610 (32.0%) |
|  | Don't know/Prefer not to answer | 121 (2.4%) |
| **Which of the following reasons for brushing the dog's teeth are the most important for you?** Enter the main reasons, maximum 3 options **(17)** | To avoid anesthesia or surgery due to dental problems | 15661 (26.1%) |
|  | To avoid veterinary cost for dental problems | 9311 (15.5%) |
|  | That the dog should keep its teeth | 39894 (66.5%) |
|  | That the vet recommends it | 2932 (4.9%) |
|  | To avoid bad breath in the dog | 15837 (26.4%) |
|  | That it is good for the dog's general health | 38641 (64.4%) |
|  | None of the above/Don't know | 5873 (9.8%) |
|  | Other reason | 1278 (2.1%) |

**Table B: Results from the veterinarian (V) and veterinary nurse (VN) survey**. Number in parenthesis after the question corresponds to the number of the question in the full survey

|  |  | |  | | **Not at all important** | | | **Not very important** | | **Sometimes important** | | **Fairly important** | | | **Very important** | | | | **Don’t know** |
| --- | --- | --- | --- | --- | --- | --- | --- | --- | --- | --- | --- | --- | --- | --- | --- | --- | --- | --- | --- |
| **What do you consider important for good dental health in dogs? (8)** *(visible to all respondents, rest of questionnaire only visible to respondents who answered yes on question 5, i.e. if they see dogs in their professional role as veterinarian/veterinary nurse)* | **Tooth brushing** | | V | | 11 (1.0%) | | | 24 (2.2%) | | 125 (11.6%) | | 155 (14.4%) | | | 712 (66.4%) | | | | 46 (4.3%) |
|  |  |  | VN | | 1 (0.2%) | | | 3 (0.5%) | | 30 (5.0%) | | 77 (12.9%) | | | 476 (79.9%) | | | | 9 (1.5%) |
|  | **Dental cleaning with textiles** | | V | | 16 (1.5%) | | | 86 (8.0%) | | 273 (25.4%) | | 325 (30.3%) | | | 249 (23.2%) | | | | 125 (11.6%) |
|  |  |  | VN | | 11 (1.9%) | | | 26 (4.4%) | | 131 (22.1%) | | 208 (35.0%) | | | 198 (33.3%) | | | | 20 (3.4%) |
|  | **Dog food special for dental health** | | V | | 84 (7.8%) | | | 336 (31.2%) | | 315 (29.3%) | | 170 (15.8%) | | | 45 (4.2%) | | | | 126 (11.7%) |
|  |  |  | VN | | 32 (5.4%) | | | 191 (32.1%) | | 224 (37.6%) | | 91 (15.3%) | | | 33 (5.5%) | | | | 24 (4.0%) |
|  | **Dental chews special for dental health** | | V | | 187 (17.4%) | | | 430 (40.0%) | | 242 (22.5%) | | 94 (8.7%) | | | 16 (1.5%) | | | | 106 (9.9%) |
|  |  |  | VN | | 128 (21.5%) | | | 234 (39.3%) | | 145 (24.3%) | | 53 (8.9%) | | | 10 (1.7%) | | | | 26 (4.4%) |
|  | **Natural chewing bones, e.g. rawhide** | | V | | 105 (9.8%) | | | 282 (26.4%) | | 272 (25.4%) | | 268 (25.1%) | | | 53 (5.0%) | | | | 89 (8.3%) |
|  |  |  | VN | | 81 (13.6%) | | | 201 (33.8%) | | 156 (26.2%) | | 103 (17.3%) | | | 32 (5.4%) | | | | 22 (3.7%) |
|  | **Dog toothpaste** | | V | | 170 (15.8%) | | | 370 (34.5%) | | 232 (21.6%) | | 144 (13.4%) | | | 66 (6.2%) | | | | 92 (8.6%) |
|  |  |  | VN | | 80 (13.5%) | | | 203 (34.2%) | | 157 (26.4%) | | 97 (16.3%) | | | 37 (6.2%) | | | | 20 (3.4%) |
|  | | | | | |  | **Very uncommon** | | **Fairly uncommon** | | **Fairly**  **common** | | | **Very common** | | | **Don't know** | | |
| **How common or uncommon do you consider dental problems to be in the dogs you meet?** Problems include calculus, gingivitis, gum disease, tooth fractures and other dental diseases and injuries. **(9)** | | | | | | V | 2 (0.2%) | | 29 (3.2%) | | 333 (37.1%) | | | 513 (57.2%) | | | 20 (2.2%) | | |
|  |  |  |  |  |  | VN | 5 (0.8%) | | 8 (1.3%) | | 195 (35.3%) | | | 342 (62.0%) | | | 2 (0.4%) | | |
| **How common or uncommon do you consider gum disease (periodontal disease) to be in the dogs you meet? (10)** | | | | | | V | 9 (1.0%) | | 132 (14.7%) | | 480 (53.5%) | | | 219 (24.4%) | | | 57 (6.4%) | | |
|  |  |  |  |  |  | VN | 2 (0.4%) | | 43 (7.8%) | | 331 (60.0%) | | | 155 (28.1%) | | | 21 (3.8%) | | |
|  | |  | | | |  | **No, never** | | **Yes, sometimes** | | **Yes, often** | | | **Yes, always** | | | | **Don't know** | |
| **Do you recommend that dog owners use any of the following to improve the dog’s dental health? (11)** | | **Tooth brushing** | | | | V | 31 (3.5%) | | 90 (10.1%) | | 198 (22.3%) | | | 553 (62.3%) | | | | 15 (1.7%) | |
|  |  |  |  |  |  | VN | 4 (0.7%) | | 31 (5.6%) | | 125 (22.7%) | | | 388 (70.5%) | | | | 2 (0.4%) | |
|  |  | **Dental cleaning with textiles** | | | | V | 142 (16.0%) | | 320 (36.0%) | | 310 (34.9%) | | | 94 (10.6%) | | | | 22 (2.5%) | |
|  |  |  |  |  |  | VN | 38 (6.9%) | | 193 (35.1%) | | 239 (43.5%) | | | 77 (14.0%) | | | | 3 (0.5%) | |
|  |  | **Dog food special for dental health** | | | | V | 425 (47.9%) | | 348 (39.2%) | | 67 (7.6%) | | | 19 (2.1%) | | | | 28 (3.2%) | |
|  |  |  |  |  |  | VN | 203 (36.9%) | | 285 (51.8%) | | 51 (9.3%) | | | 6 (1.1%) | | | | 5 (0.9%) | |
|  |  | **Dental chews special for dental health** | | | | V | 489 (55.2%) | | 294 (33.2%) | | 73 (8.2%) | | | 10 (1.1%) | | | | 20 (2.3%) | |
|  |  |  |  |  |  | VN | 305 (55.5%) | | 192 (34.9%) | | 40 (7.3%) | | | 4 (0.7%) | | | | 9 (1.6%) | |
|  |  | **Natural chewing bones, e.g. rawhide** | | | | V | 439 (49.5%) | | 306 (34.5%) | | 107 (12.1%) | | | 14 (1.6%) | | | | 21 (2.4%) | |
|  |  |  |  |  |  | VN | 323 (58.7%) | | 174 (31.6%) | | 41 (7.5%) | | | 9 (1.6%) | | | | 3 (0.5%) | |
|  |  | **Dog toothpaste** | | | | V | 217 (24.5%) | | 404 (45.6%) | | 187 (21.1%) | | | 60 (6.8%) | | | | 19 (2.1%) | |
|  |  |  |  |  |  | VN | 91 (16.5%) | | 274 (49.8%) | | 149 (27.1%) | | | 32 (5.8%) | | | | 4 (0.7%) | |
|  | | | |  | | | | | | | | | **V** | | | **VN** | | | |
| **When do you provide information about dental cleaning (with toothbrush or textiles) to dog owners?** Several options can be specified **(14)** | | | | Puppy vaccination | | | | | | | | | 364 (44.2%) | | | 316 (58.6%) | | | |
|  |  |  |  | Other routine visit (e.g. vaccination) | | | | | | | | | 581 (70.5%) | | | 426 (79.0%) | | | |
|  |  |  |  | Visit for dental cleaning (calculus removal) | | | | | | | | | 631 (76.6%) | | | 448 (83.1%) | | | |
|  |  |  |  | Visit due to dental problems | | | | | | | | | 655 (79.5%) | | | 426 (79.0%) | | | |
|  |  |  |  | Visit due to other disease | | | | | | | | | 421 (51.1%) | | | 193 (35.8%) | | | |
|  |  |  |  | Special information meeting | | | | | | | | | 42 (5.1%) | | | 75 (13.9%) | | | |
|  |  |  |  | Don't know/Prefer not to answer | | | | | | | | | 23 (2.8%) | | | 11 (2.0%) | | | |
|  |  |  |  | Other | | | | | | | | | 61 (7.4%) | | | 29 (5.4%) | | | |
| **How do you provide information about dental cleaning (with toothbrush or textiles) to dog owners?** Several options can be specified **(15)** | | | | Verbally | | | | | | | | | 789 (96.2%) | | | 520 (96.7%) | | | |
|  |  |  |  | Written | | | | | | | | | 191 (23.3%) | | | 181 (33.6%) | | | |
|  |  |  |  | Practical demonstration | | | | | | | | | 258 (31.5%) | | | 253 (47.0%) | | | |
|  |  |  |  | Information about web pages or similar | | | | | | | | | 53 (6.5%) | | | 38 (7.1%) | | | |
|  |  |  |  | Don't know/Prefer not to answer | | | | | | | | | 13 (1.6%) | | | 6 (1.1%) | | | |
|  |  |  |  | Other | | | | | | | | | 7 (0.9%) | | | 10 (1.9%) | | | |
| **What home dental cleaning frequency do you recommend? (16)** | | | | Daily | | | | | | | | | 570 (69.6%) | | | 402 (74.9%) | | | |
|  |  |  |  | Every other day | | | | | | | | | 40 (4.9%) | | | 25 (4.7%) | | | |
|  |  |  |  | Once a week | | | | | | | | | 50 (6.1%) | | | 12 (2.2%) | | | |
|  |  |  |  | As often as they have time for | | | | | | | | | 75 (9.2%) | | | 64 (11.9%) | | | |
|  |  |  |  | I don't specify | | | | | | | | | 29 (3.5%) | | | 10 (1.9%) | | | |
|  |  |  |  | Don't know/Prefer not to answer | | | | | | | | | 10 (1.2%) | | | 4 (0.8%) | | | |
|  |  |  |  | Other | | | | | | | | | 45 (5.5%) | | | 20 (3.7%) | | | |
| **Do you follow up whether the dog owner is satisfactorily performing dental home care on the dog?** Follow up means checking if the dog owner is carrying out dental home care on the dog, e.g., via telephone call, email, visit or re-visit**. (17)** | | | | No, never | | | | | | | | | 332 (38.2%) | | | 196 (36.1%) | | | |
|  |  |  |  | Yes, sometimes | | | | | | | | | 391 (45.0%) | | | 262 (48.3%) | | | |
|  |  |  |  | Yes, often | | | | | | | | | 76 (8.8%) | | | 54 (9.9%) | | | |
|  |  |  |  | Yes, always | | | | | | | | | 10 (1.2%) | | | 8 (1.5%) | | | |
|  |  |  |  | Don't know/Prefer not to answer | | | | | | | | | 60 (6.9%) | | | 23 (4.2%) | | | |
| **What do you consider the most common reasons why veterinary health practitioners do not inform dog owners about tooth brushing?** Maximum 3 options can be specified **(24)** | | | | Lack of time | | | | | | | | | 436 (50.8%) | | | 302 (56.2%) | | | |
|  |  |  |  | Considered unimportant | | | | | | | | | 91 (10.6%) | | | 28 (5.2%) | | | |
|  |  |  |  | Dog owner could take offence | | | | | | | | | 29 (3.4%) | | | 16 (3.0%) | | | |
|  |  |  |  | Dog behaviour expected to make tooth brushing impossible | | | | | | | | | 204 (23.8%) | | | 160 (29.8%) | | | |
|  |  |  |  | Dog owner not expected to manage tooth brushing on dog | | | | | | | | | 153 (17.8%) | | | 90 (16.8%) | | | |
|  |  |  |  | Occasion not suitable | | | | | | | | | 272 (31.7%) | | | 223 (41.5%) | | | |
|  |  |  |  | Forgets | | | | | | | | | 391 (45.6%) | | | 218 (40.6%) | | | |
|  |  |  |  | Don´t know/Prefer not to answer | | | | | | | | | 111 (12.9%) | | | 52 (9.7%) | | | |
|  |  |  |  | Other reason | | | | | | | | | 27 (3.1%) | | | 22 (4.1%) | | | |
| **What do you consider to be the most common reason for dog owners not to brush their dog's teeth? (23)** | | | | Dog unwilling/difficult to handle | | | | | | | | | 232 (26.9%) | | | 128 (23.7%) | | | |
|  |  |  |  | Lack of knowledge | | | | | | | | | 341 (39.6%) | | | 283 (52.4%) | | | |
|  |  |  |  | Lack of time | | | | | | | | | 103 (11.9%) | | | 49 (9.1%) | | | |
|  |  |  |  | Dog owner considers tooth brushing unimportant | | | | | | | | | 103 (11.9%) | | | 62 (11.5%) | | | |
|  |  |  |  | Don't know/Prefer not to answer | | | | | | | | | 51 (5.9%) | | | 6 (1.1%) | | | |
|  |  |  |  | Other | | | | | | | | | 32 (3.7%) | | | 12 (2.2%) | | | |

**Table C. Questions related to the construct “Veterinary health practitioners’ attitudes towards dental chews and dental feed”**. Number in parenthesis after the question corresponds to the number of the question in the full survey.

| **“Veterinary health practitioners’ attitudes towards dental chews and dental feed” *(ChewFeed)*** (Cronbach’s alpha 0.80) |
| --- |
| Do you consider special dental chews, which according to the manufacturer benefit dental health, to be important for good dental health in dogs? (8) |
| Do you consider special dog food, which according to the manufacturer benefits dental health, to be important for good dental health in dogs? (8) |
| Do you consider chewing bones (e.g. rawhide, bully sticks, pig ears or pig tails) to be important for good dental health in dogs? (8) |
| Do you consider chewing toys (e.g. squeaky toys, textile toys, artificial bones, stuffed toys) to be important for good dental health in dogs? (8) |
| Do you recommend that dog owners use special dental chews, which according to the manufacturer benefit dental health, to improve the dog’s dental health? (11) |
| Do you consider dog tooth paste to be important for good dental health in dogs? (8) |
| Do you recommend that dog owners use special dog food, which according to the manufacturer benefits dental health, to improve the dog’s dental health? (11) |
| Do you recommend that dog owners use chewing bones (e.g. rawhide, bully sticks, pig ears or pig tails) to improve the dog’s dental health? (11) |

**Table D. Background characteristics of dogs**

| **Year of birth^a^** |  |
| --- | --- |
| 2003 or earlier | 1183 (2.0%) |
| 2004-2007 | 6804 (11.3%) |
| 2008-2011 | 13274 (22.1%) |
| 2012-2013 | 11865 (19.8%) |
| 2014-2015 | 17687 (29.5%) |
| 2016-2017 | 9066 (15.1%) |
| Don’t know^b^ | 99 (0.2%) |
| **Breed of dog^a^** | |
| Group 1: Sheepdogs and Cattledogs (except Swiss Cattledogs) | 6760 (11.3%) |
| Group 2: Pinscher and Schnauzer - Molossoid and Swiss Mountain and Cattledogs | 6476 (10.8%) |
| Group 3: Terriers | 5601 (9.3%) |
| Group 4: Dachshunds | 1329 (2.2%) |
| Group 5: Spitz and primitive types | 5274 (8.8%) |
| Group 6: Scent hounds and related breeds | 2494 (4.2%) |
| Group 7: Pointing Dogs | 1733 (2.9%) |
| Group 8: Retrievers - Flushing Dogs - Water Dogs | 10639 (17.7%) |
| Group 9: Companion and Toy Dogs | 8968 (15.0%) |
| Group 10: Sighthounds | 1044 (1.7%) |
| Mixed breed | 9178 (15.3%) |
| Other breed | 407 (0.7%) |
| Don’t know^b^ | 73 (0.1%) |
| **Weight** | |
| 0 - 2.9 kg | 1979 (3.3%) |
| 3 - 5.9 kg | 6661 (11.1%) |
| 6 - 9.9 kg | 10941 (18.2%) |
| 10 - 14.9 kg | 8296 (13.8%) |
| 15 - 19.9 kg | 7305 (12.2%) |
| 20 - 29.9 kg | 13761 (22.9%) |
| 30 kg or more | 10885 (18.1%) |
| Don’t know^b^ | 150 (0.3%) |
| **Sex** | |
| Female | 24803 (41.4%) |
| Male | 21895 (36.5%) |
| Female, neutered | 4514 (7.5%) |
| Male, neutered | 8753 (14.6%) |
| Don’t know^b^ | 13 (0.0%) |

*^a^Merged into groups by authors*

*^b^Option also includes “prefer not to answer” in addition to “don’t know”*

**Table E. Background characteristics of dog owners**

| **Year of birth^a^** | |
| --- | --- |
| 1946 or earlier | 3587 (6.0%) |
| 1947-1966 | 25683 (43.1%) |
| 1967-1986 | 24517 (41.1%) |
| 1987 or later | 5758 (9.6%) |
| Don’t know^b^ | 103 (0.2%) |
| **Gender** | |
| Woman | 44579 (74.8%) |
| Man | 14659 (24.6%) |
| Prefer not to answer/Other | 392 (0.7%) |
| **County^c^** | |
| Urban county (Stockholm, Skåne, Västra Götaland) | 27441 (46.1%) |
| Rural county (Other) | 31909 (53.6%) |
| Don’t know^b^ | 186 (0.3%) |
| **Main employment^a^** | |
| Student | 2087 (3.5%) |
| Employed | 34935 (58.7%) |
| Self-employed | 6660 (11.2%) |
| Retired | 8714 (14.6%) |
| Other | 6232 (10.5%) |
| Don’t know^b^ | 862 (1.4%) |
| **Highest level of education** | |
| Not completed primary school | 110 (0.2%) |
| Elementary school or equivalent | 3096 (5.2%) |
| Gymnasium | 17069 (28.7%) |
| Vocational training | 8658 (14.5%) |
| University or college less than three years | 8790 (14.8%) |
| University or college three years or more | 20251 (34.1%) |
| Don’t know^b^ | 1439 (2.4%) |
| **Healthcare profession^d^** | |
| Yes | 13434 (22.6%) |
| No | 43185 (72.7%) |
| Prefer not to answer/Other | 2746 (4.6%) |
| **Dog breeder** | |
| Yes | 5030 (8.4%) |
| No | 54425 (91.1%) |
| Don’t know^b^ | 266 (0.4%) |

*^a^Merged into groups by authors*

*^b^Option also includes “prefer not to answer” in addition to “don’t know”*

*^c^Municipalities merged into counties by authors*

*^d^Healthcare professions: Assistant nurse, Nurse, Physician, Dental nurse, Dental hygienist, Dentist, Animal caretaker, Veterinary nurse, Veterinarian*

**Table F: Background characteristics of veterinarians and veterinary nurses**. V denotes veterinarians and VN denotes veterinary nurses.

| **Year of birth^a^** | **V (%)** | **VN (%)** |
| --- | --- | --- |
| 1950 or earlier | 74 (6.6%) | 0 (0%) |
| 1951-1960 | 139 (12.5%) | 39 (6.4%) |
| 1961-1970 | 171 (15.4%) | 127 (20.9%) |
| 1971-1980 | 277 (24.9%) | 208 (34.2%) |
| 1981-1993 | 451 (40.5%) | 234 (38.4%) |
| Don’t know^b^ | 2 (0.2%) | 1 (0.2%) |
| **Year of degree in veterinary medicine/nursing^a,^ ^c^** | | |
| 1999 or earlier | 376 (33.8%) | 51 (8.4%) |
| 2000-2011 | 375 (33.7%) | 176 (28.9%) |
| 2012-2017 | 360 (32.3%) | 357 (58.6%) |
| Don’t know^b^ | 3 (0.3%) | 25 (4.1%) |
| **Gender** | | |
| Female | 854 (76.7%) | 592 (97.2%) |
| Male | 249 (22.4%) | 12 (2.0%) |
| Prefer not to answer/Other | 11 (1.0%) | 5 (0.8%) |
| **County^d^** | | |
| Urban county (Stockholm, Skåne, Västra Götaland) | 690 (61.9%) | 311 (51.1%) |
| Rural county (Other) | 424 (38.1%) | 298 (48.9%) |
| Don’t know^b^ | 0 (0.0%) | 0 (0%) |
| **See dogs in their professional role as veterinarian/veterinary nurse** | | |
| No, never | 172 (15.4%) | 34 (5.6%) |
| Yes, sometimes | 245 (22.0%) | 24 (3.9%) |
| Yes, often | 687 (61.7%) | 543 (89.1%) |
| Don’t know^b^ | 10 (0.9%) | 8 (1.3%) |
| **Work in a pet clinic or animal hospital for dogs, cats and smaller animals** *(only visible to respondents who answered yes on previous question)* | | |
| Yes | 684 (73.2%) | 544 (95.9%) |
| No | 240 (25.7%) | 21 (3.7%) |
| Don’t know^b^ | 10 (1.1%) | 2 (0.4%) |
| **Size of the pet clinic/animal hospital^a^** *(only visible to respondents who answered yes on previous question)* | | |
| 1-2 veterinarians | 165 (24.2%) | 85 (15.6%) |
| 3-10 veterinarians | 332 (48.6%) | 261 (47.9%) |
| 11 veterinarians or more | 174 (25.5%) | 195 (35.8%) |
| Don’t know^b^ | 12 (1.8%) | 4 (0.7%) |

*^a^Merged into groups by authors*

*^b^Option also includes “prefer not to answer” in addition to “don’t know”*

*^c^In 2000 the first Veterinary nurses with a two-year University education received their degree (yrkesexamen) in Sweden. In 2009 the education was prolonged to three years*

*^d^Municipalities merged into counties by author*
