# Supplementary material for: Dental home care in dogs - a questionnaire study among Swedish dog owners, veterinarians and veterinary nurses
Source: BMC Vet Res. 2020 Mar 18;16:90. doi: 10.1186/s12917-020-02281-y (PMC7081671; doi:10.1186/s12917-020-02281-y)
Supplement: Supplementary file 2 — Additional file 2: Figure S1. File containing supplementary figures. A. Odds ratio (95% CI) for dog owners’ background characteristics influence on brushing frequency. B. Odds ratio (95% CI) for dog owners’ background characteristics influence on perceived reception of recommendations at the veterinary clinic to brush their dog’s teeth. C. Odds ratio (95% CI) for veterinarians’ and veterinary nurses’ background characteristics influence on stating tooth brushing to be important to good dental health in dogs. D. Associations between background characteristics of veterinarians/veterinary nurses, and their attitude towards dental chews and dental feed. E. Odds ratio of the influence of administration of dental chews on brushing the dogs teeth. F. Construct’s “Dog owners’ attitudes towards brushing dogs’ teeth” (BrushAttitude) association with the year of birth of the dog and dog owner. G. Construct’s “Veterinary health practitioners’ attitudes towards dental chews and dental feed” (ChewFeed) and Construct’s “Veterinary health practitioners’ attitudes and opinions on dental problems and dental cleaning” (Cleaning) association with the year of degree of the veterinary health practitioner. [file 12917_2020_2281_MOESM2_ESM.pdf]

Dental Home Care in Dogs - a questionnaire study among Swedish Dog Owners, Veterinarians and Veterinary Nurses

Supplementary Information Figures

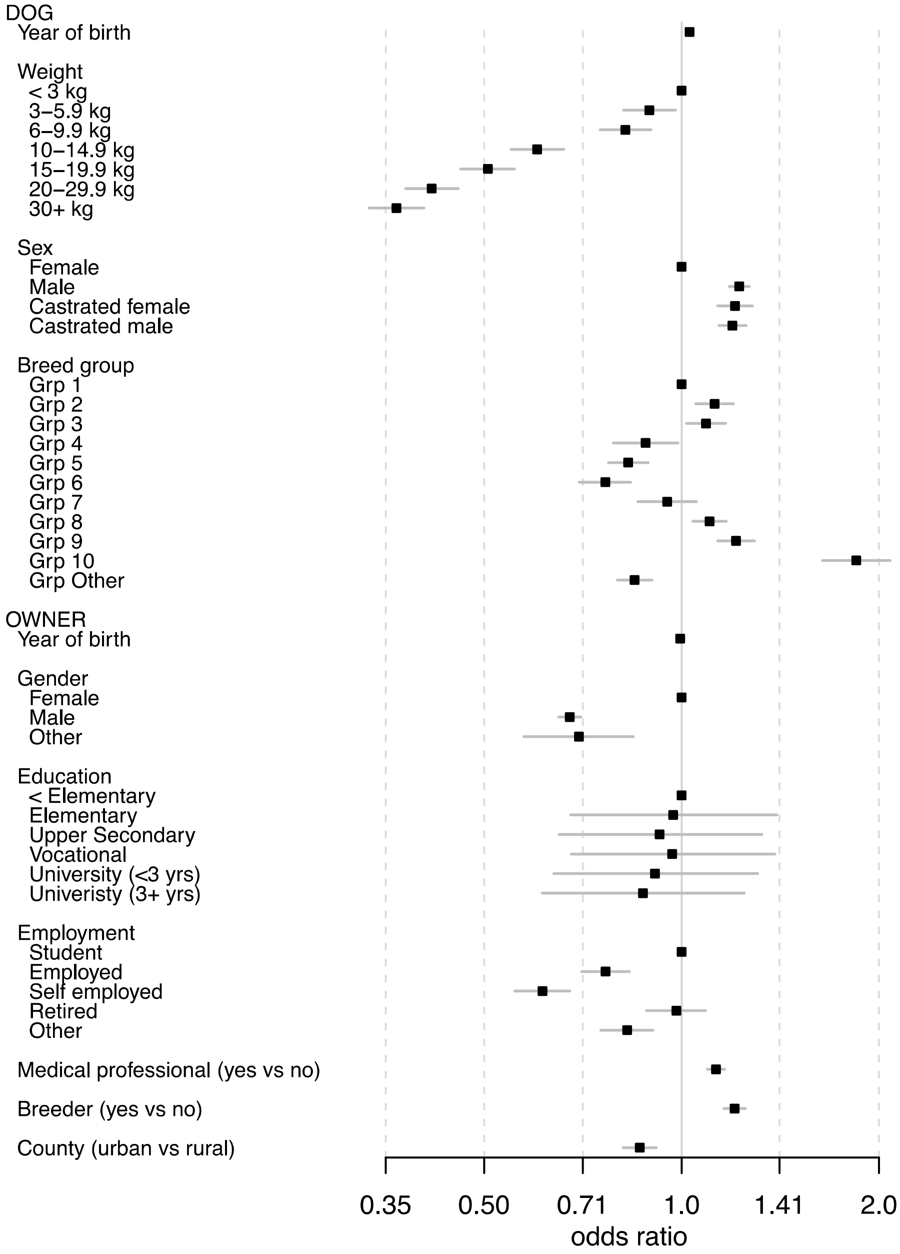

Fig A. Dog owners: How often in the last month have you brushed your dog's teeth with a toothbrush? (14). Odds ratio (95% CI) for dog owners' background characteristics influence on brushing frequency.

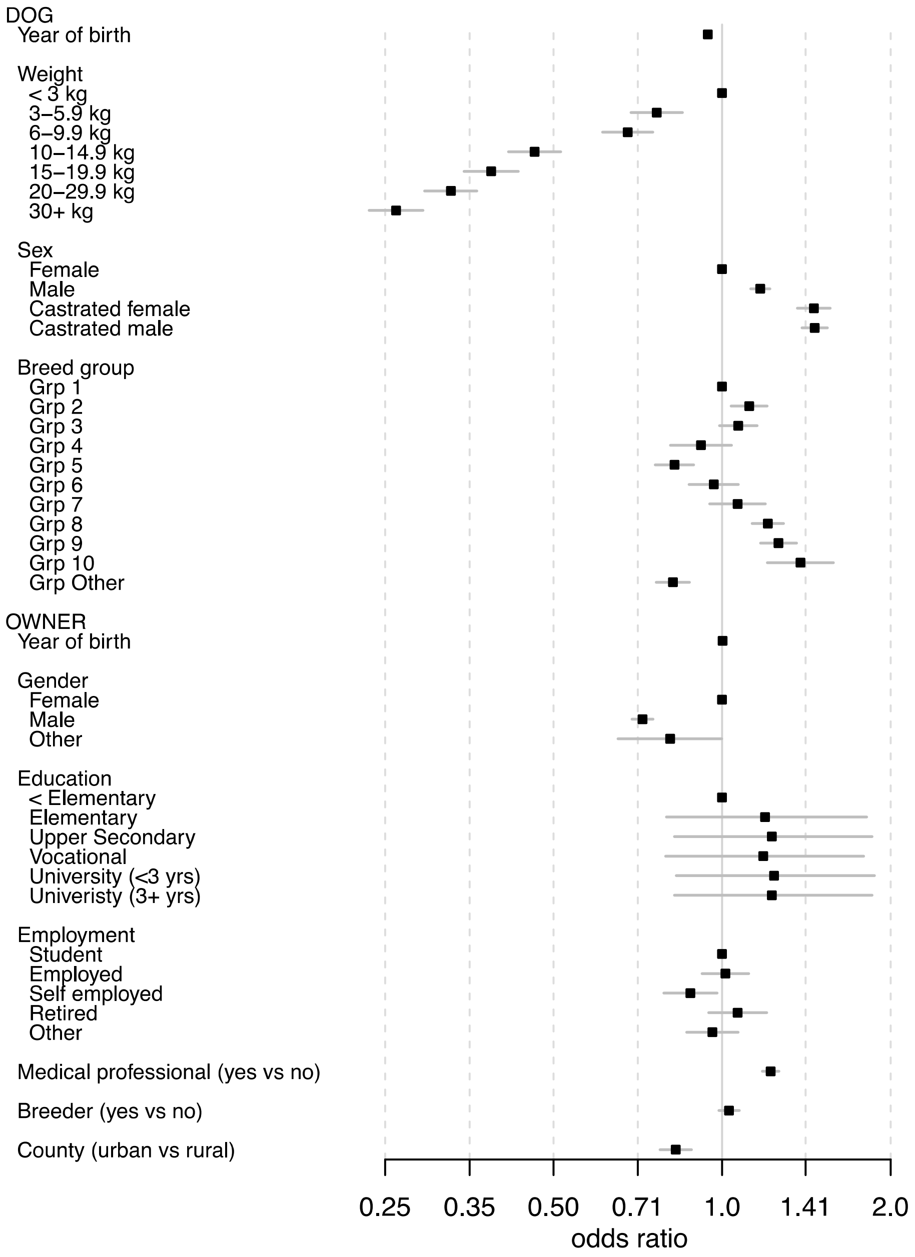

Fig B. Dog owners: Have you been advised at a veterinary clinic to use tooth brushing to improve your dog's dental health? (27). Odds ratio (95% CI) for dog owners' background characteristics influence on perceived reception of recommendations at the veterinary clinic to brush their dog's teeth.

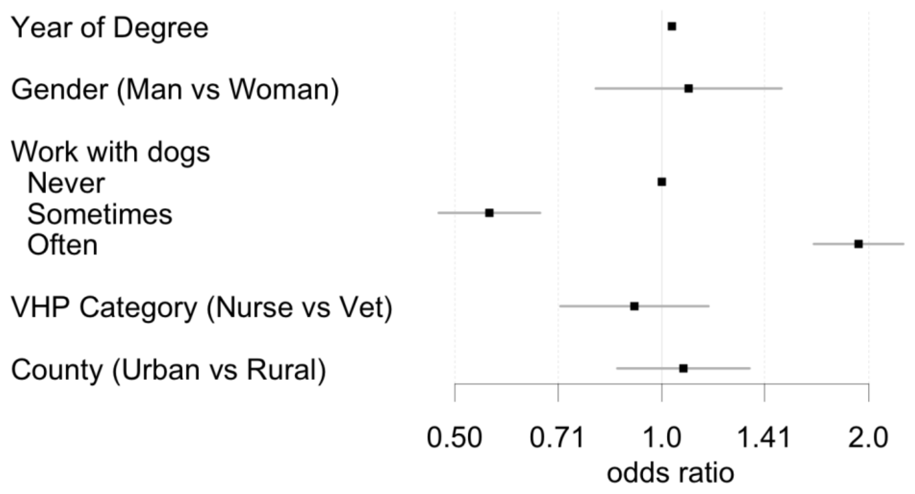

Fig C. Veterinarians and veterinary nurses: What do you consider important for good dental health in dogs? Tooth brushing (8). Odds ratio (95% CI) for veterinarians' and veterinary nurses' background characteristics influence on stating tooth brushing to be important to good dental health in dogs.

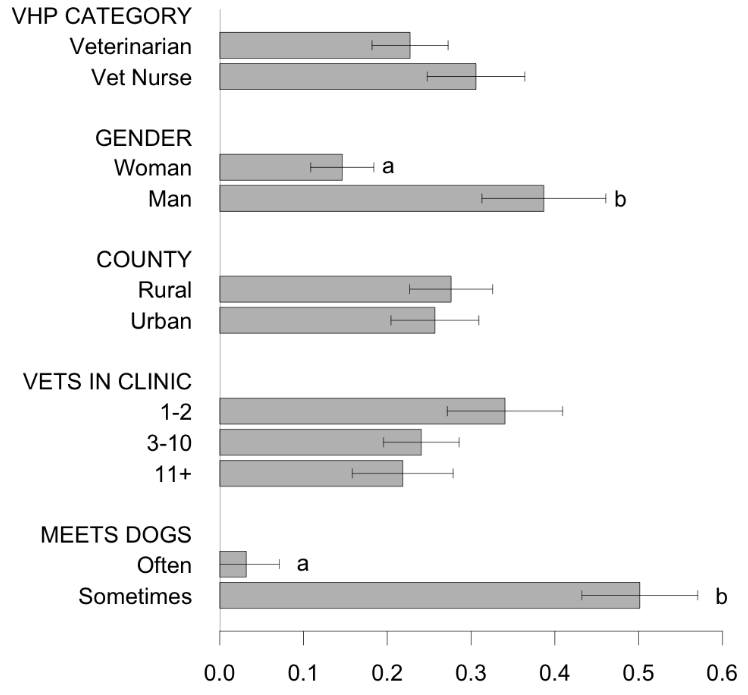

Fig D. Construct "Veterinary health practitioners' attitudes towards dental chews and dental feed" (ChewFeed). Associations between background characteristics of veterinarians/veterinary nurses, and their attitude towards dental chews and dental feed, where a higher construct score reflects a more positive attitude towards dental chews and dental feed. Scores should only be compared within figure. (Note that negative scores do not automatically reflect a negative attitude towards dental chews and dental feed).

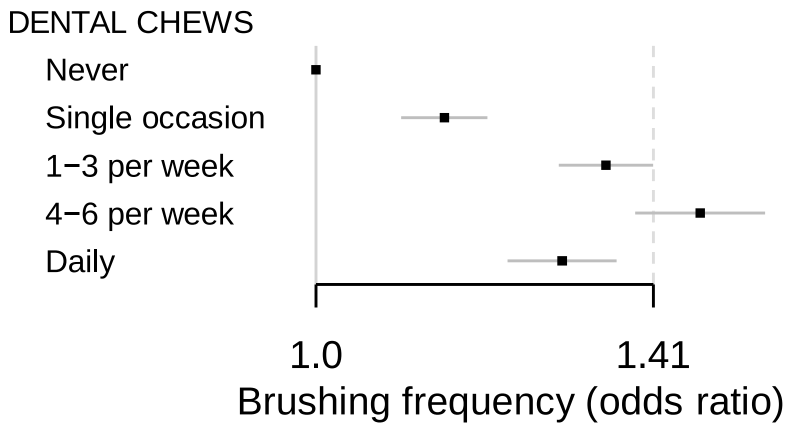

Fig E. Odds ratio of the influence of administration of dental chews on brushing the dogs teeth.

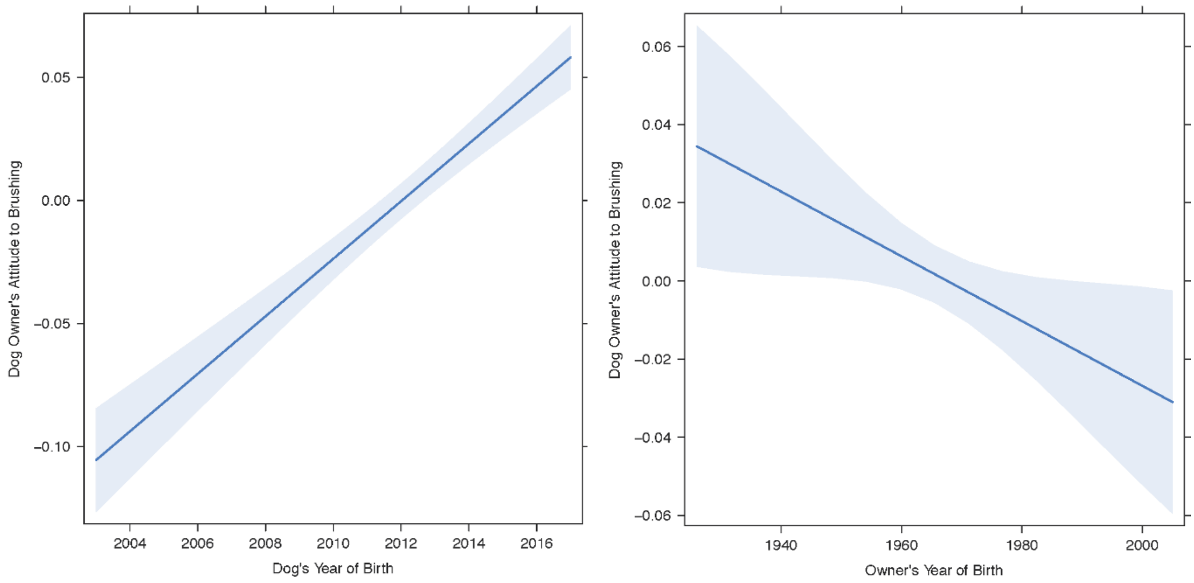

Fig F. Construct's "Dog owners' attitudes towards brushing dogs' teeth" (BrushAttitude) association with the year of birth of the dog (left) and dog owner (right). A higher construct score reflects a more positive attitude towards tooth brushing. (Note that negative scores do not automatically reflect a negative attitude).

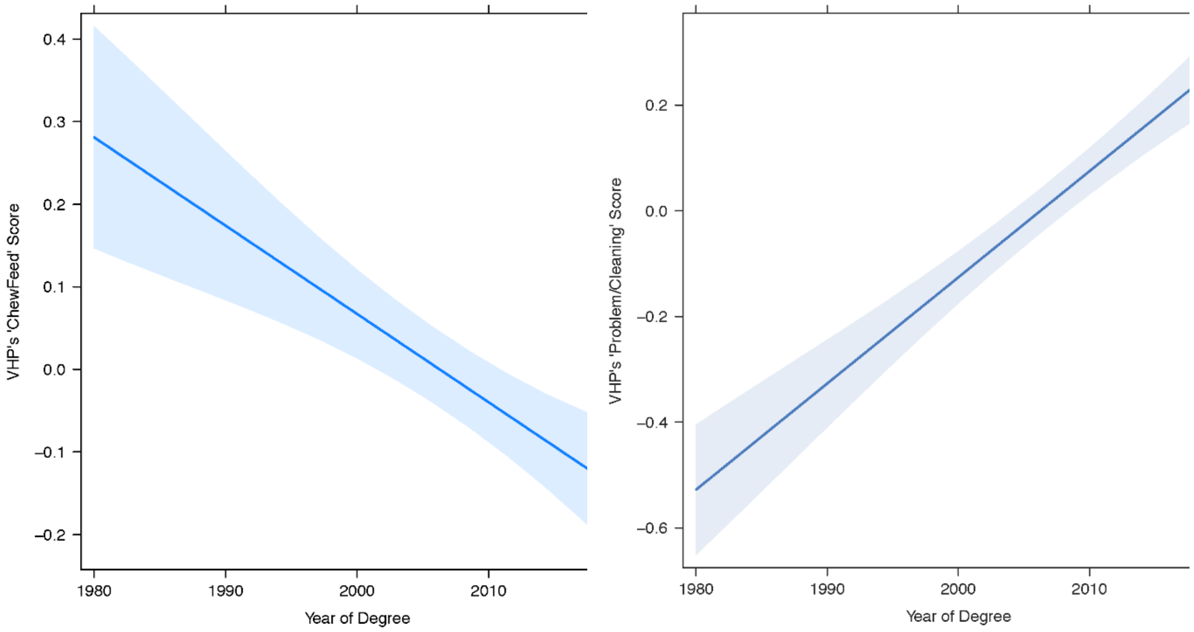

Fig G. Constructs' "Veterinary health practitioners' attitudes towards dental chews and dental feed" (ChewFeed)(left) and "Veterinary health practitioners' attitudes and opinions on dental problems and dental cleaning" (Cleaning)(right) association with the year of degree of the veterinary health practitioner. A higher construct score reflects a more positive attitude towards dental chews/feed (left) respective dental problems and dental cleaning/tooth brushing (right). (Note that negative scores do not automatically reflect a negative attitude).
